# Successful direct-acting antiviral therapy improves circulating mucosal-associated invariant T cells in patients with chronic HCV infection

**DOI:** 10.1371/journal.pone.0244112

**Published:** 2020-12-31

**Authors:** Apichaya Khlaiphuengsin, Natthaya Chuaypen, Pimpayao Sodsai, Rangsima Reantragoon, Win Min Han, Anchalee Avihingsanon, Pisit Tangkijvanich

**Affiliations:** 1 Center of Excellence in Hepatitis and Liver Cancer, Department of Biochemistry, Faculty of Medicine, Chulalongkorn University, Bangkok, Thailand; 2 Center of Excellence in Immunology and Immune-mediated Diseases, Department of Microbiology, Faculty of Medicine, Chulalongkorn University, Bangkok, Thailand; 3 HIV Netherlands Australia Thailand Research Collaboration (HIV-NAT), The Thai Red Cross AIDS Research Center, Bangkok, Thailand; Kaohsiung Medical University Chung Ho Memorial Hospital, TAIWAN

## Abstract

**Objectives:**

Mucosal-associated invariant T (MAIT) cells have been shown to contribute in the pathogenesis of various liver diseases, including chronic hepatitis C virus (HCV) infection. This study was aimed at investigating the frequency, phenotype, and function of circulating MAIT cells, as well as their alterations after successful direct-acting antivirals (DAAs) in HCV-infected patients with or without HIV infection.

**Methods:**

A total 85 patients (51 HCV-monoinfection and 34 HCV/HIV-coinfection), who received elbasvir/grazoprevir from a clinical trial and 20 healthy controls were included. MAIT cells in blood were characterized using flow cytometry at baseline and 24 weeks post-treatment.

**Results:**

HCV-monoinfected and HCV/HIV-coinfected patients achieved similar sustained virological response rates (SVR24, 94.1% vs. 97.1%). Circulating MAIT cells in the monoinfection and coinfection groups were presented at low frequencies in comparison with healthy controls (median, 1.1% vs. 1.1% vs. 2.4%, *P*<0.001) and exhibited features of chronic activation and impaired functional capacity. A negative correlation between circulating MAIT cell frequency and liver stiffness assessed by magnetic resonance elastography was observed. Compared with baseline, increased in circulating MAIT cells after successful DAA therapy was mainly detected in HCV-monoinfected patients compared with HCV/HIV-coinfected individuals. Moreover, MAIT cell restoration was predominantly observed among patients with significant fibrosis to cirrhosis (F2-F4).

**Conclusions:**

These data indicated that dysregulation of MAIT cells might play a role in the progression of chronic HCV infection. Partial restoration of MAIT cell frequency and function was observed after successful DAA therapy, particularly in HCV-monoinfected patients.

## Introduction

Chronic hepatitis C virus (HCV) infection represents one of the leading causes of chronic hepatitis, cirrhosis and hepatocellular carcinoma (HCC) worldwide [1]. Due to sharing route of transmission, coinfection with human immunodeficiency virus (HIV) is common in HCV-infected individuals. Moreover, HCV/HIV-coinfection is associated with accelerated risk for cirrhosis and liver-related complications compared to monoinfection [2]. The current standard of care for chronic HCV infection is direct-acting antivirals (DAAs), which provides sustained virological response (SVR) rates over 90% [3]. Successful HCV eradication remarkably reduces progressive liver disease and improves extrahepatic manifestations [3]. However, data regarding immune restoration following DAA therapy in HCV-infected patients with or without HIV are incomplete and needed additional investigations.

It is well-recognized that persistent stimulation by HCV antigens during chronic infection is associated with activation and impaired immunological functions of natural killer (NK) cells and HCV-specific T cells [4]. Recently, mucosal-associated invariant T (MAIT) cells, another immune cell population, have gained increasing attention due to their enrichment in the liver and peripheral blood [5]. MAIT cells are innate-like T cells that characterized by the co-expressing an invariant Vα7.2-Jα33 T cell receptor (TCR) [5]. These cells represent approximately 20–50% of intrahepatic T cells and 1–10% of circulating T cells in healthy individuals. MAIT cells play an essential role in antimicrobial response by the interaction between their TCR and riboflavin-derivative metabolites presented by the major histocompatibility complex (MHC) class I‐related protein 1 (MR1) [6]. These cells can also be activated in a TCR-independent manner by pro-inflammatory cytokines interleukin (IL)-12 and IL-18 during chronic viral infection [7]. Upon activation, MAIT cells rapidly secret various pro-inflammatory cytokines including interferon-gamma (IFN-γ) and upregulate cytotoxic molecules such as granzyme B contributing to inflammatory processes.

The dominance of MAIT cells in the liver indicates that they might play an essential role in pathogenesis of various chronic liver diseases (CLD), including HCV [8]. Among HCV-infected patients, the frequencies and functions of intrahepatic and peripheral MAIT cells are significantly diminished as compared with healthy controls [9]. Moreover, it was recently shown in Western populations that impaired peripheral MAIT cells did not recover after successful antiviral therapy [10–12]. Despite these data, it is unclear whether the restoration of circulating MAIT cells might occur upon HCV eradication in other ethnic populations. To address this important issue, we conducted a study to characterize circulating MAIT cells in a large cohort of Thai patients with HCV-monoinfection and HCV/HIV-coinfection. We also investigated the alteration of MAIT cells following successful DAA therapy. Our results demonstrated that elimination of HCV by DAAs could, to some extent, restore MAIT cell frequency and function, especially in monoinfected individuals.

## Methods

### Patients

Thai patients with HCV genotype 1, who were treated with elbasvir/grazoprevir (EBR/GZR) (clinicaltrials.gov; NCT03037151) at the King Chulalongkorn Memorial Hospital, Bangkok, Thailand between August 2018 and June 2019 were recruited. Inclusion criteria for therapy were patients with anti-HCV positive≥6 months with HCV RNA levels>10,000 IU/mL. All HCV/HIV-coinfected patients received long-term antiretroviral therapy (ART) with undetectable plasma HIV-RNA at enrollment. Treatment-naïve patients received EBR/GZR for 12 weeks, while individuals previously treated with pegylated interferon and ribavirin (RBV) were assigned to receive EBR/GZR plus RBV for 16 weeks. Exclusion criteria were coinfection with hepatitis B virus (HBV), heavy alcohol consumption, previous DAA treatment, decompensated cirrhosis and HCC. Healthy individuals without conditions affecting MAIT cells were recruited as controls.

Liver stiffness (LS) was assessed at baseline and week 24 post-treatment by magnetic resonance elastography (MRE) using MR imaging system Philips Ingenia at 3.0 T (Philips Healthcare, Best, the Netherlands) [13]. LS was measured in three slices and an average value expressed in kilopascals (kPa) was obtained. The cut-off values for significant fibrosis (≥F2), advanced fibrosis (≥F3) and cirrhosis (F4) were 3.2, 4.0 and 4.6 kPa, respectively [14].

The study protocol was approved by the Institutional Review Board, Faculty of Medicine, Chulalongkorn University (IRB No.281/62) with written informed consent obtained from all participants, and the study was conducted according to the Helsinki Declaration and Good Clinical Practice guidelines.

### PBMCs isolation and staining by flow cytometry

Peripheral blood mononuclear cells (PBMC) were isolated by Ficoll-Hypaque density gradient centrifugation using Percoll PLUS density gradient media (GE Healthcare, Philadelphia, PA, USA) at 400 rcf (xg) for 30 minutes at room temperature. The obtained PBMCs were washed twice and stored in liquid nitrogen.

Approximately 500,000 viable PBMCs were incubated with/without 25 ng/mL of IL‐12 and 50 ng/mL of IL‐18 (Biolegend, San Diego, CA, USA) in RPMI 1640 supplemented with 10% FBS, 1X GlutaMAX, 1X MEM non-essential amino acids (MEM NEAA), 20 mM HEPES, 1 mM sodium pyruvate, 100 U/mL penicillin, and 100 μg/mL streptomycin (GIBCO, New York, NY, USA) for a total of 24 hours at 37°C at 5% CO_2_. Brefeldin A (10 μg/mL, Sigma) was added 5 hours prior to labelling cells with anti‐human CD3‐PE/Cy7 (clone UCHT1); anti‐human CD4‐APC/Cy7 (clone RPAT4); anti‐human CD8‐PE/Cy5 (clone HIT8a); anti‐human CD161‐BV421 (clone HP‐3G10); anti‐human TCR Vα7.2‐PE (clone 3C10), anti‐human CD69‐AF700 (cloneFN50), anti‐human CD38‐APC (clone HB7); anti‐human PD1‐BV510 (clone H4A3); anti‐human TIM3-BV785 (clone F38-2E2) and anti‐human CTLA4-BV605 (clone BNI3)and (Biolegend, San Diego, CA, USA). Subsequently, cells were fixed, permeabilized and stained with anti‐IFN-γ‐APC (4S.B3), anti‐TNF-α‐BV650 (Mab11), anti‐CD107a‐BV510 (H4A3) (Biolegend, San Diego, CA, USA) and anti‐granzyme‐B‐AF700 (GB11) (BD Biosciences, San Jose, CA, USA). Cells were washed, measured by CytoFLEX flow cytometry (CytoFLEX, Beckman Coulter, Indianapolis, IN, USA) and analysed using Flowjo software version 10 (Tree star, Ashland, Oregon, USA).

### Statistical analysis

Statistical analysis was performed using SPSS version 22 (SPSS Inc., Chicago, IL, USA) and GraphPad Prism v8.0 (GraphPad Software, San Diego, CA) software. One-Way ANOVA, followed by Bonferroni's comparison pos-hoc test, Kruskal-Wallis test, followed by a multiple comparison test, t-test, Wilcoxon signed-rank test or Mann-Whitney U test were applied as appropriate. Data correlation were analyzed by Spearman’s rank test. A *P* value <0.05 was considered statistically significant.

## Results

### Baseline demographic and clinical characteristics

A total of 85 patients (51 HCV-monoinfection and 34 HCV/HIV-coinfection) and 20 healthy controls (HC) were enrolled (Table 1). The average ages of patients with HCV/HIV-coinfection and HC were comparable but younger than that of the monoinfection group. BMI in the monoinfection group and HC was similar but higher than that of the coinfection group. The rates of SVR24 in HCV-monoinfection and HCV/HIV-coinfection were comparable [94.1%(48/51) vs. 97.1%(33/34),*P* = 0.647].

**Table 1:** Baseline demographic and clinical characteristics.

Characteristics	Healthy controls (n = 20)	HCV-monoinfected patients (n = 51)	HCV-HIV-coinfected patients (n = 34)	*P-*value
Age, years	40.0(38.3–48.0)	50.0(41.0–58.0)	42.5(35.8–46.5)	**<0.001**
Sex, male	12(60.0)	37(72.5)	28(82.4)	0.197
BMI, kg/m^2^	24.0(21.1–25.9)	24.1(22.7–27.5)	22.4(20.1–23.6)	**0.019**
Treatment outcome (SVR24)				0.647
SVR		48(94.1)	33(97.1)	
Non-SVR		3(5.9)	1(2.9)	
Hemoglobin, g/dL		14.2(13.2–15.1)	15.4(13.9–16.0)	0.199
White blood cells, 10^3^/L		5.6(4.6–6.8)	5.9(4.9–6.5)	0.782
CD4+ T-cell count, cells/mm^3^			502.7(390.5–613.7)	
CD8+ T-cell count, cells/mm^3^			523.0(231.0–523.0)	
CD4+/CD8+ ratio			0.7(0.6–0.7)	
Platelets, 10^3^/L		187.0(151.0–256.0)	187.0(157.0–245.5)	0.785
AST, U/L	21.0(19.0–24.5)	43.0(31.0–57.0)	39.5(30.0–55.3)	**0.023**
ALT, U/L	24.0(18.5–32.5)	53.0(34.0–77.0)	43.5(30.8–83.8)	0.119
eGFR, mL/min/1.73m^2^		101.0(86.1–109.8)	98.0(86.0–108.4)	0.744
Log_10_ HCV RNA, IU/mL		6.4(5.9–6.7)	6.5(6.0–6.8)	0.989
HIV RNA suppression (<50 copies/mL)			34(100)	
FIB-4 index		0.4(0.3–0.7)	0.3(0.2–0.6)	0.174
Liver stiffness (kPa)		3.0(2.6–3.9)	2.7(2.4–3.4)	0.344
Fibrosis score				0.174
F0-F1		19(37.2)	21(61.8)	
F2		16(31.4)	7(20.6)	
F3		8(15.7)	3(18.8)	
F4		8(15.7)	3(18.8)	

Data presented as numbers (%) or median (Interquartile range, IQR). Statistical analysis; Chi-squared test, t-test or ANOVA as appropriate. BMI; Body mass index, SVR; sustained virological response, Aspartate aminotransferase, ALT; Alanine aminotransferase, Cr; Creatinine, eGFR; Estimated glomerular filtration rate

### Characterization of circulating MAIT cells at baseline

**Fig 1:**
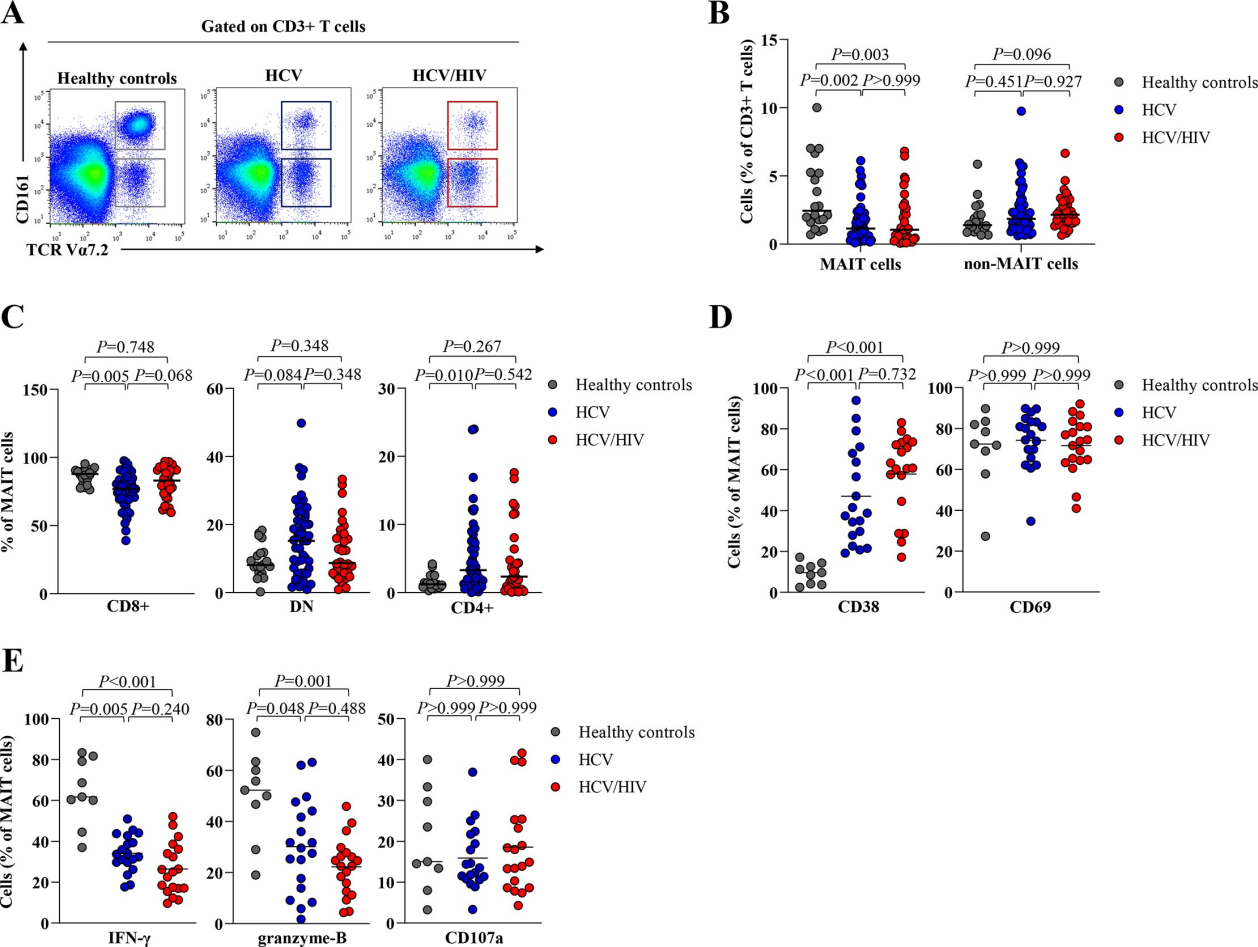
Frequency of MAIT cells and non-MAIT cells in healthy controls and HCV-infected patients. (A) Representative dot plots for identification of TCRVα7.2+CD161++ or MAIT cells and TCRα7.2+ CD161− or non-MAIT cells. FSC-A, forward scatter area; SSC-A, side scatter area. (B) Percentage of MAIT and non-MAIT cells in blood of healthy controls, HCV-monoinfected patients (HCV) and HCV/HIV-coinfected patients (HCV/HIV). (C) Percentage of CD8+, CD4+ and double negative (DN) MAIT cell subsets in blood. (D) Frequency of CD38+ and CD69+ MAIT cells. (E) Frequency of polyfunctionality; IFN-γ, cytolytic function marker; granzyme-B and cytotoxicity marker; CD107a-positive MAIT cells after overnight stimulation with IL-12/IL18. Data presented as median and interquartile range (IQR). Statistical analysis was performed using Kruskal–Wallis, followed by a multiple comparison test.

We determined MAIT cells, identified as CD3+TCRVα7.2+CD161++ in blood samples of HC and the patient groups (Fig 1A). The full gating strategy is displayed in S1 Fig. A significant lower median of total MAIT cells was detected in the patient groups compared to HC (HC = 2.4%,IQR = 1.7–5.2%, HCV = 1.1%,IQR = 0.5–2.0%,*P* = 0.002 and HCV/HIV = 1.1%,IQR = 0.4–2.7%, *P* = 0.003). In contrast, no difference of non-MAIT cells (CD3+ TCRVα7.2+ CD161-) were observed among groups (Fig 1B). Compared to HC, the monoinfection group exhibited lower CD8+ MAIT cells (HC = 87.9%,IQR = 81.0–90.0% vs. HCV = 76.9%,IQR = 69.1–84.1%,*P* = 0.005) but higher CD4+ MAIT cells (HC = 1.2%,IQR = 0.8–2.3% vs. HCV = 3.3%,IQR = 1.3–7.6%,*P* = 0.010) (Fig 1C). However, there was no significant difference in total MAIT cells and their sub-populations between the patient groups (Fig 1B and 1C).

Compared to HC, the frequency of CD38 MAIT cells, a marker of chronic activation, were significantly higher in HCV-infected patients (HC = 9.7%,IQR = 3.9–13.5%, HCV = 38.7%, IQR = 27.9–68.0%,*P*<0.001 and HCV/HIV = 60.8%,IQR = 44.5–73.3%,*P*<0.001). However, there was no difference in CD69 expressing MAIT cells among groups (Fig 1D). Compared to HC, HCV-infected patients had significantly lower IFN-γ (HC = 61.7%,IQR = 52.3–80.4%, HCV = 33.7%,IQR = 29.6–42.7%,*P* = 0.005 and HCV/HIV = 25.0%,IQR = 16.9–36.1%,*P*<0.001) and granzyme-B (HC = 52.2%,IQR = 37.9–61.7%, HCV = 29.6%,IQR = 13.9–44.1%,*P* = 0.048 and HCV/HIV = 22.5%,IQR = 12.6–27.8%,*P* = 0.001) (Fig 1E), but there was no difference in the levels of PD-1, TIM-3 and CTLA-4-positive MAIT cells. (S2 Fig).

**Fig 2:**
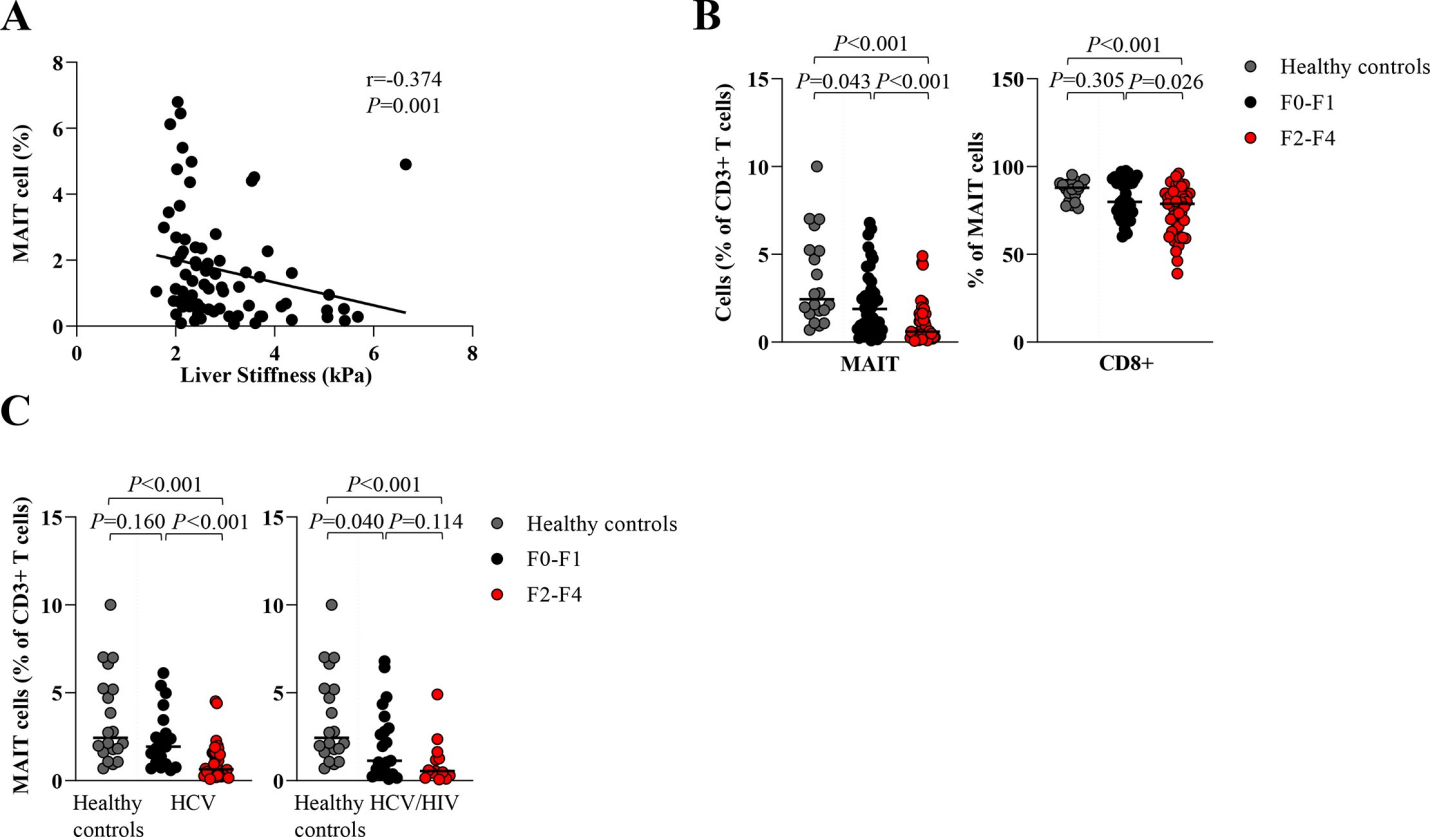
Circulating MAIT and CD8+ MAIT cells according to liver fibrosis stages. (A) Correlation between frequency of MAIT cells and liver stiffness. (B) Percentage of total MAIT cells and CD8+ MAIT cells in patients with no to mild fibrosis (F0-F1) and significant fibrosis to cirrhosis (F2-F4) compared to healthy controls. (C) Percentage of total MAIT cells in HCV-monoinfected patients (HCV) and HCV/HIV-coinfected patients (HCV/HIV) with F0-F1 and F2-F4 compared to healthy controls. Data presented as median and interquartile range (IQR). Correlations were assessed by Spearman's rank test (A). Statistical analysis was performed using Mann-Whitney test (B-C).

Additionally, we found a negative correlation between total MAIT cells and LS (r = -0.374,*P* = 0.003) (Fig 2A). However, there was no correlation between the frequencies of MAIT cells and HCV RNA (r = 0.056, *P* = 0.616). A significant reduction of MAIT cells was observed in the F2-F4 group compare to the F0-F1 group (0.6%, IQR = 0.3–1.6% vs. 11.9%, IQR = 0.8–3.3%, *P*<0.001). The reduction of CD8+MAIT cells was also detected in the corresponding groups (78.8%,IQR = 63.8–84.4% vs. 79.9%,IQR = 74.1–92.3%,*P* = 0.026) (Fig 2B). Regarding HIV status, significant difference of total MAIT cells was observed in the monoinfection group on the basis of different fibrosis stages (F2-F4 = 0.7%,IQR = 0.3–1.6% vs. F0-F1 = 1.9%,IQR = 0.9–3.5%,*P* = 0.001). There was a similar trend among HCV/HIV-coinfection patients (F2-F4 = 0.6%,IQR = 0.2–1.4% vs. F0-F1 = 1.1%,IQR = 0.5–3.3%,*P* = 0.114) (Fig 2C).

### Changes in circulating MAIT cell frequency after therapy

**Fig 3:**
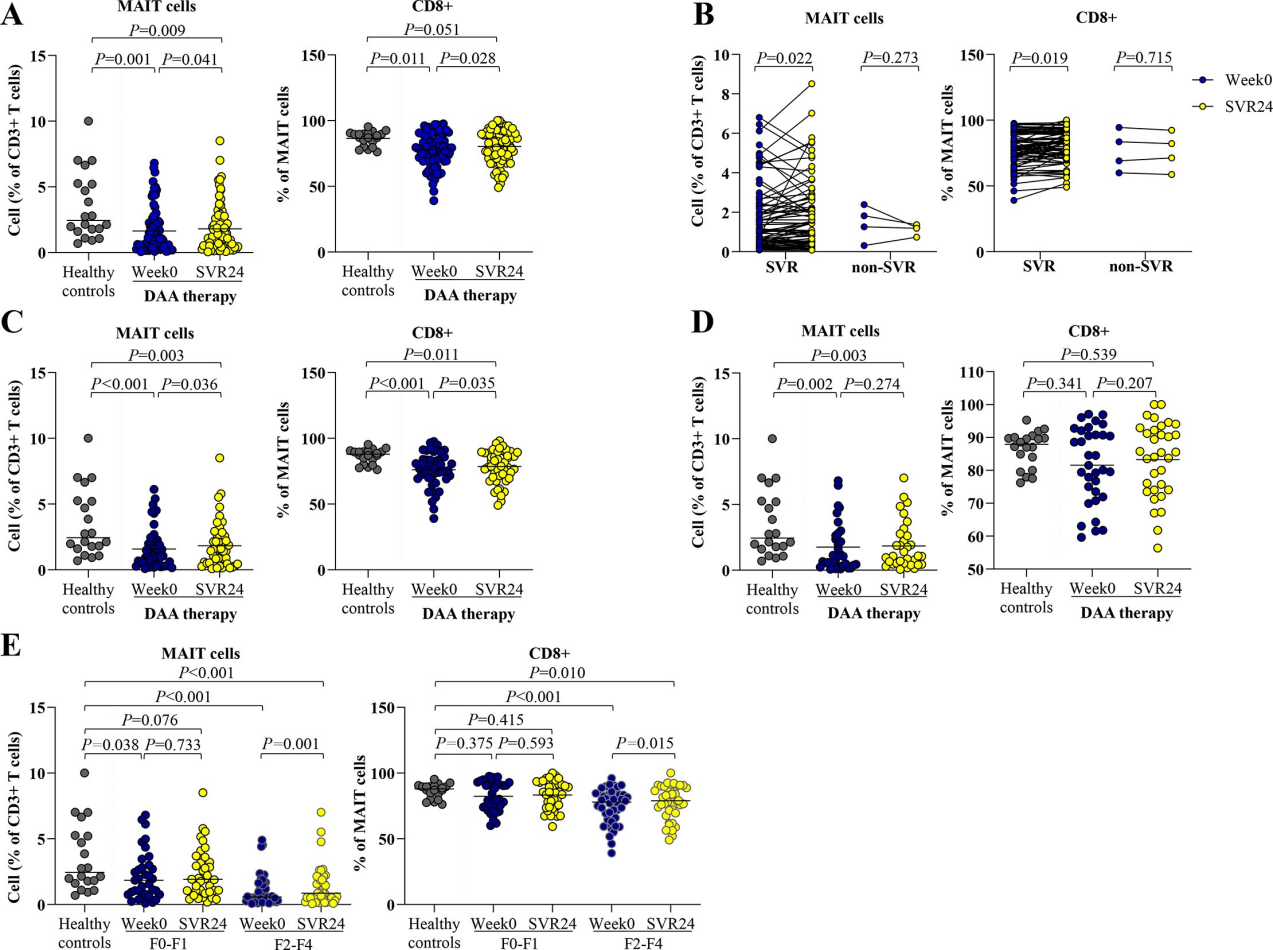
Circulating MAIT and CD8+ MAIT cells in blood of HCV-infected patients at pre- and post-treatment. (A) Percentage of MAIT cells and CD8+ MAIT cells at pre-treatment (week0) and post-treatment (SVR24) in all HCV-infected patients compared to healthy controls. (B) Percentage of MAIT cells and CD8+ MAIT cells in patients achieving sustained virological response (SVR) and non-SVR. (C) Percentage of MAIT cells and CD8+ MAIT cells in HCV-monoinfected patients achieving SVR. (D) Percentage of MAIT cells and CD8+ MAIT cells in HCV/HIV-coinfected patients achieving SVR. (E) Percentage of MAIT cells and CD8+ MAIT cells according to liver fibrosis stages. Data presented as median and IQR. Statistical comparison was tested was tested using Mann-Whitney test for comparing two independent samples or Wilcoxon signed-rank test for comparing paired data samples (A-E).

At post-treatment, the overall cohort had a significant increase in levels of MAIT cells (week 0 = 1.1%,IQR = 0.4–2.3% vs. SVR24 = 1.4%,IQR = 0.5–2.6%,*P* = 0.041) and CD8+ MAIT cells (week 0 = 79.5%,IQR = 70.4–90.1% vs. SVR24 = 82.8%,IQR = 72.5–90.2%,*P* = 0.028), although they did not reach the levels of HC (MAIT = 2.4%,IQR = 1.7–5.2% and CD8+ MAIT = 87.9%,IQR = 81.0–90.0%) (Fig 3A). These changes were observed only in responders achieving SVR24 but were not detected in non-responders (Fig 3B).

Subsequently, only 81 responders (48 HCV-monoinfection and 33 HCV/HIV-coinfection) were further analyzed. Among these responders, MAIT cells (week0 = 1.1%,IQR = 0.4–2.3% vs. SVR24 = 1.4%,IQR = 0.5–2.6%,*P* = 0.022) and CD8+ MAIT cells (week0 = 79.5%,IQR = 70.8–90.1% vs. SVR24 = 82.9%,IQR = 73.2–90.2%,*P* = 0.019) were significantly increased at post-treatment (Fig 3B). Conversely, CD4+ MAIT cells were significantly decreased compared with baseline (*P* = 0.003) (S3 Fig). Notably, only HCV-monoinfected patients displayed significant alterations of MAIT cells (week0 = 1.1%,IQR = 0.5–2.0% vs. SVR24 = 1.4%,IQR = 0.5–2.6%,*P* = 0.036) and CD8+ MAIT cells (week0 = 77.0%,IQR = 69.5–84.6% vs. SVR24 = 79.4%,IQR = 70.7–89.2%,*P* = 0.035) (Fig 3C). However, such significant differences were not detected in the coinfection group (MAIT cells: week0 = 1.1%,IQR = 0.4–2.7% vs. SVR24 = 1.1%,IQR = 0.5–2.7%,*P* = 0.274; CD8+ MAIT cells: week0 = 81.5%,IQR = 72.7–91.5% vs. SVR24 = 85.4%,IQR = 74.1–92.6%,*P* = 0.207) (Fig 3D).

Regarding liver fibrosis, the F2-F4 group had a higher change of MAIT cells (week0 = 0.6%,IQR = 0.3–1.6% vs. SVR24 = 0.9,IQR = 0.3–1.9%,*P* = 0.001) than the F0-F1 group (week0 = 1.8%,IQR = 0.8–3.5% vs. SVR24 = 1.9,IQR = 1.0–3.6%,*P* = 0.733). The alteration of CD8+ MAIT cells after successful therapy was detected in the respective groups (week0 = 78.0%, IQR = 64.2–84.2% vs. SVR24 = 79.0, IQR = 71.0–89.1%, *P* = 0.015 and week0 = 80.2%, IQR = 74.1–92.3% vs. SVR24 = 85.4, IQR = 74.1–92.9%, *P* = 0.593) (Fig 3E).

In subgroup analysis, HCV-monoinfected patients with F2-F4 exhibited significant changes of MAIT cells (week0 = 0.7%, IQR = 0.3–1.6% vs. SVR24 = 0.9, IQR = 0.3–1.9%, *P* = 0.009) and CD8+ MAIT cells (week0 = 77.0%, IQR = 63.3–83.7% vs. SVR24 = 78.4, IQR = 69.1–88.8%, *P* = 0.011). In the coinfection group with F2-F4, a significant change in MAIT cells was also observed (week0 = 0.5%, IQR = 0.2–1.5% vs. SVR24 = 0.6, IQR = 0.3–2.1%, *P* = 0.040) but there was no significant alteration in CD8+ MAIT cells (week0 = 79.9%, IQR = 65.2–87.7% vs. SVR24 = 81.6, IQR = 72.6–90.8%, *P* = 0.273).

Notably, post-treatment LS reduction was negatively correlated with increased MAIT cells (r = -0.255, *P* = 0.043) (S4 Fig). Among HCV-monoinfected patients who achieved SVR24, a significant change of LS value after therapy was observed (week0 = 2.9, IQR = 2.6–3.9 kPa vs. SVR24 = 2.7, IQR = 2.4–3.6 kPa, *P* = 0.012) Additionally, the change in LS value was negatively correlated with MAIT cell alteration in this subgroup of patients (r = -0.312, *P* = 0.031).

### Functional alterations of circulating MAIT cells after successful therapy

**Fig 4:**
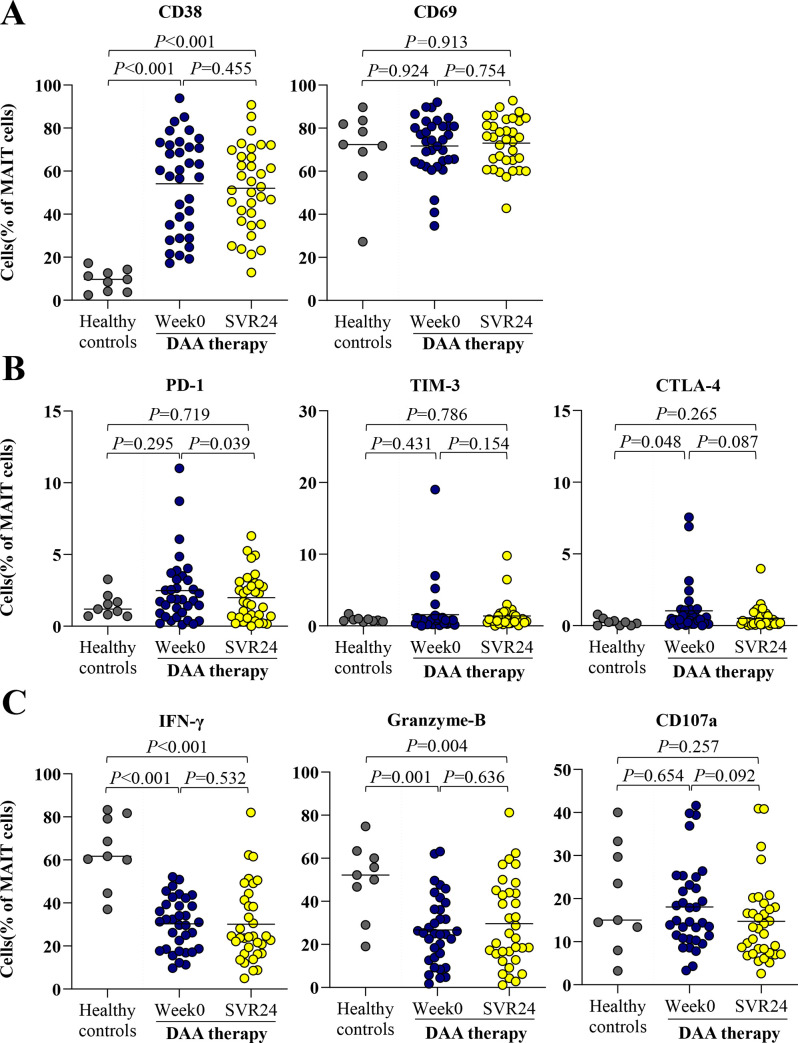
Activation, exhaustion and function of MAIT cells healthy controls and HCV-infected patients at pre- and post-treatment. (A) Frequency of CD38 and CD69 MAIT cells at pre-treatment (week0) and post-treatment (SVR24). (B) Expression of exhaustion markers, PD-1, TIM-3 and CTLA-4 MAIT cells. (C) Expression of polyfunctionality; IFN-γ, cytolytic function marker; granzyme-B and cytotoxicity marker; CD107a-positive MAIT cells after overnight stimulation with IL-12/IL18. Data presented as median and IQR. Statistical comparison was tested using Mann-Whitney test for comparing two independent samples or Wilcoxon signed-rank test for comparing paired data samples.

We further investigated whether MAIT cell activation, exhaustion and cytokine production were altered after successful DAA therapy. As shown in Fig 4A, no significant change of activation markers, CD38 and CD69, were observed in responders. Among exhaustion markers, responders exhibited a significant reduction of PD-1-positive MAIT cells (week0 = 1.9%%,IQR = 0.8–3.3% vs. SVR24 = 1.7%,IQR = 0.6–2.9%,*P* = 0.039) and had a trend toward decreasing CTLA-4-positive MAIT cells (week0 = 0.4%%,IQR = 0.2–1.1% vs. SVR24 = 0.3%,IQR = 0.1–0.7%,*P* = 0.087) (Fig 4B). The frequencies of cytokine production by MAIT cells after stimulated with IL12/IL-18 in responders did not change after DAA therapy (Fig 4C).

## Discussion

Recent advances indicate that dysregulation of MAIT cells is an essential factor contributing to the development of various CLD [5]. In the cross-sectional study, we examined the frequency, phenotype and function, as well as clinical relevance of circulating MAIT cells in HCV-infected patients with or without HIV infection. Additionally, longitudinal analysis allowed us to identify the changes in MAIT cells after DAA therapy. Our data indicated that MAIT cell frequency in infected individuals was significantly diminished and their levels were negatively correlated with LS. Moreover, MAIT cells showed features of chronic activation with reduced functional capacity, producing less IFN-γ upon stimulation. Dissimilar to previous reports, we found that circulating MAIT cell frequency and functions were, to some extent, reversible after successful therapy. Together, these findings suggest that dysregulation of MAIT cells is involved in the pathogenesis of chronic HCV infection, which could be partially restored after viral eradication.

In agreement with previous reports [7, 9–12, 15–18], our data confirmed that the percentages of circulating MAIT cells, particularly for CD8+ MAIT cells were significantly lower in HCV-infected patients than that in healthy controls, while the percentage of non-MAIT cells were comparable between groups. Indeed, the reduction of MAIT cells was also observed in other chronic viral hepatitis including HBV and hepatitis delta virus (HDV) [19, 20]. Although the mechanism is not yet completely elucidated, it is suggested that peripheral MAIT cell depletion might be linked to their recruitment into the liver where these immunological cells are involved in persistent inflammatory processes [9]. Notably, chronic HIV infection also exhibits peripheral MAIT cell reduction, which might partially be restored after effective ART [21]. In this study, HCV-monoinfected and HCV/HIV-coinfected patients displayed comparable levels of MAIT cells and CD8+ MAIT cells, consistently with previous reports [15, 16, 18].

Emerging evidence has identified intrahepatic MAIT cells as a novel modulator of liver fibrogenesis [22]. In this context, it has been shown that there is an inverse correlation between intrahepatic MAIT cell frequency and histologically documented fibrosis [9]. A recent report in autoimmune hepatitis indicated that intrahepatic MAIT cells promoted liver fibrosis through activation of hepatic stellate cells [23]. Moreover, activated MAIT cells were capable of stimulating pro-fibrogenic properties of myofibroblasts in models of fatty liver diseases [22]. In this study, we revealed that circulating MAIT cells was negatively associated with LS assessed by MRE and their alteration following DAA therapy was significantly correlated with LS improvement. Thus, it is anticipated that MAIT cells also display a pathogenetic role that contributes to the progression of liver fibrosis in chronic HCV infection. In this context, the reciprocal change of MAIT cell frequency and LS reduction could be attributable to an increase in MAIT cells along with the resolution of liver inflammation and fibrosis after successful antiviral therapy [9].

At present, it is unclear whether circulating MAIT cells in HCV-infected individuals could recover after successful therapy as several studies in Western countries reported an irreversible decrease of MAIT cells after HCV eradication [10–12]. However, the aforementioned studies included small sample sizes with various therapeutic regimens. We have reported herein for the first time that the frequency of circulating MAIT cells of HCV-infected patients is significantly increased after successful DAA therapy, although it has not fully restored to normal levels. Notably, HCV-monoinfected patients exhibited a better improvement of MAIT cells compared to HCV/HIV-coinfected individuals. Additionally, the restoration of MAIT cells was predominantly observed among patients with significant fibrosis (F2-F4) compared to individuals with less fibrosis stages (F0-F1). Moreover, exhaustion markers including PD-1 and CTLA-4 in MAIT cells were partially recovered, suggesting that immune responses involving MAIT cells could improve after achieving SVR.

These findings were in part consistent with previous data demonstrating that intrahepatic MAIT cells was significant restored within 4 weeks during DAA therapy, although the improvement of circulating MAIT cell frequency was not yet detected [9]. It is conceivable that MAIT cells have a low proliferation rate [24] and thus a longer duration might be necessary for a complete restoration of circulating MAIT cells. Similarly, patients with chronic HBV infection achieving HBeAg clearance exhibited a higher MAIT-cell frequency than those without the antigen clearance [20]. Additionally, it was shown that HBV-infected patients displayed decreased MAIT-cell activation markers after antiviral therapy [25]. Together, these observations suggest that depletion and function of circulating MAIT cells in patients with chronic viral hepatitis might potentially be a reversible process. Whether long-term HCV eradication could gradually reverse abnormalities in MAIT cells needs further longitudinal study. In this respect, it was recently shown that long-term and complete alcohol abstinence could significantly restore MAIT cell depletion over time in patients with alcoholic liver disease [26].

This study had some limitations as it had a relatively short duration of follow-up. To this end, further study to assess long-term impact of EBR/GZR on MAIT cell frequency and phenotype in our cohort is currently under investigation. Second, pre-treatment liver biopsy was not performed in this study as it is an invasive procedure and might not be applicable for assessing fibrosis improvement after therapy. Instead, monitoring changes of fibrosis using the reliable imaging modality such as MRE is more feasible [27]. Interestingly, it has been shown in a recent meta-analysis that LS declines significantly over time after 1–6 months of achieving SVR in patients treated with DAAs [28]. Despite these observations, however, it remains to be determined whether LS improvement in relatively short time represents true fibrosis reversal or rather rapid resolution of necroinflammatory activities, and thus might overestimate fibrosis regression [29].

In summary, our data indicated that dysregulation of MAIT cells might play a role in the progression of chronic HCV infection. Additionally, partial restoration of MAIT cell frequency and function was observed after successful DAA therapy, particularly in HCV-monoinfected patients.

## Supporting information

S1 Fig Gating strategy for identification of TCRVα7.2+CD161++ or MAIT cells and TCRα7.2+ CD161− or non-MAIT cells. (TIF)

S2 Fig Frequency of exhaustion marker; PD-1, TIM-3 and CTLA-4-positive MAIT cells. (TIF)

S3 Fig Percentage of CD4+ MAIT cells in patients achieving Sustained Virological Response (SVR) and non-SVR. (TIF)

S4 Fig Correlation between change in MAIT cells and change in liver stiffness. (TIF)
