# Supplementary figures and images for: Successful direct-acting antiviral therapy improves circulating mucosal-associated invariant T cells in patients with chronic HCV infection
Source: PLoS One. 2020 Dec 31;15(12):e0244112. doi: 10.1371/journal.pone.0244112 (PMC7775079; doi:10.1371/journal.pone.0244112)

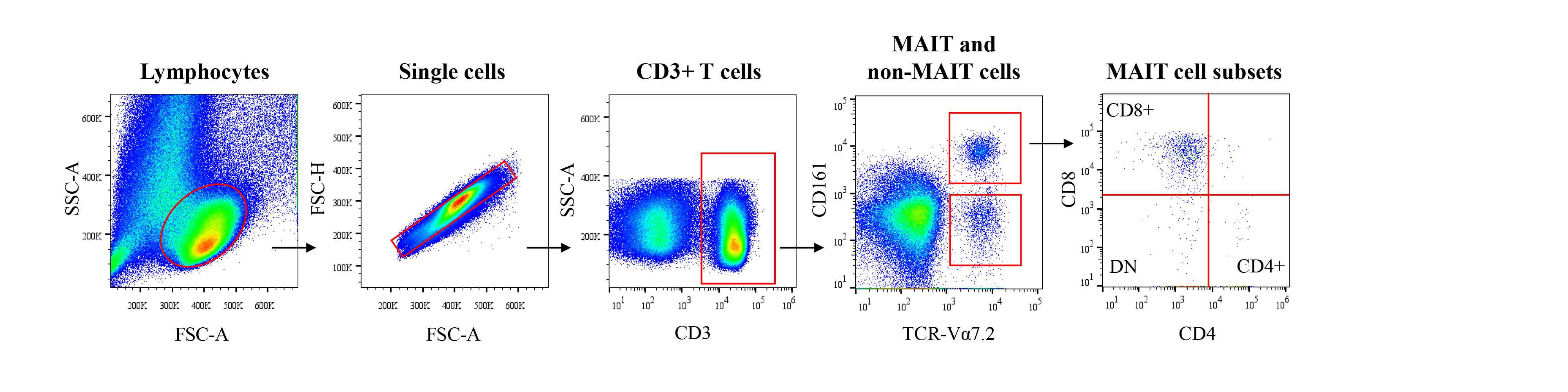

Supplement: S1 Fig — (TIF) [file pone.0244112.s001.tif]

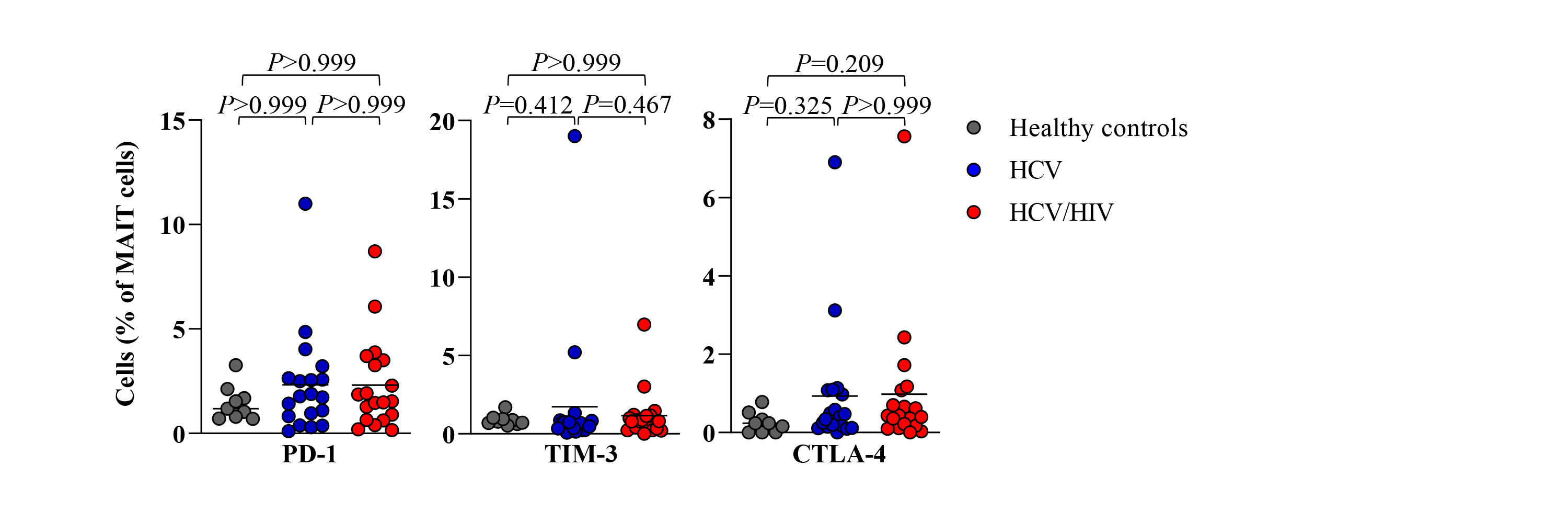

Supplement: S2 Fig — (TIF) [file pone.0244112.s002.tif]

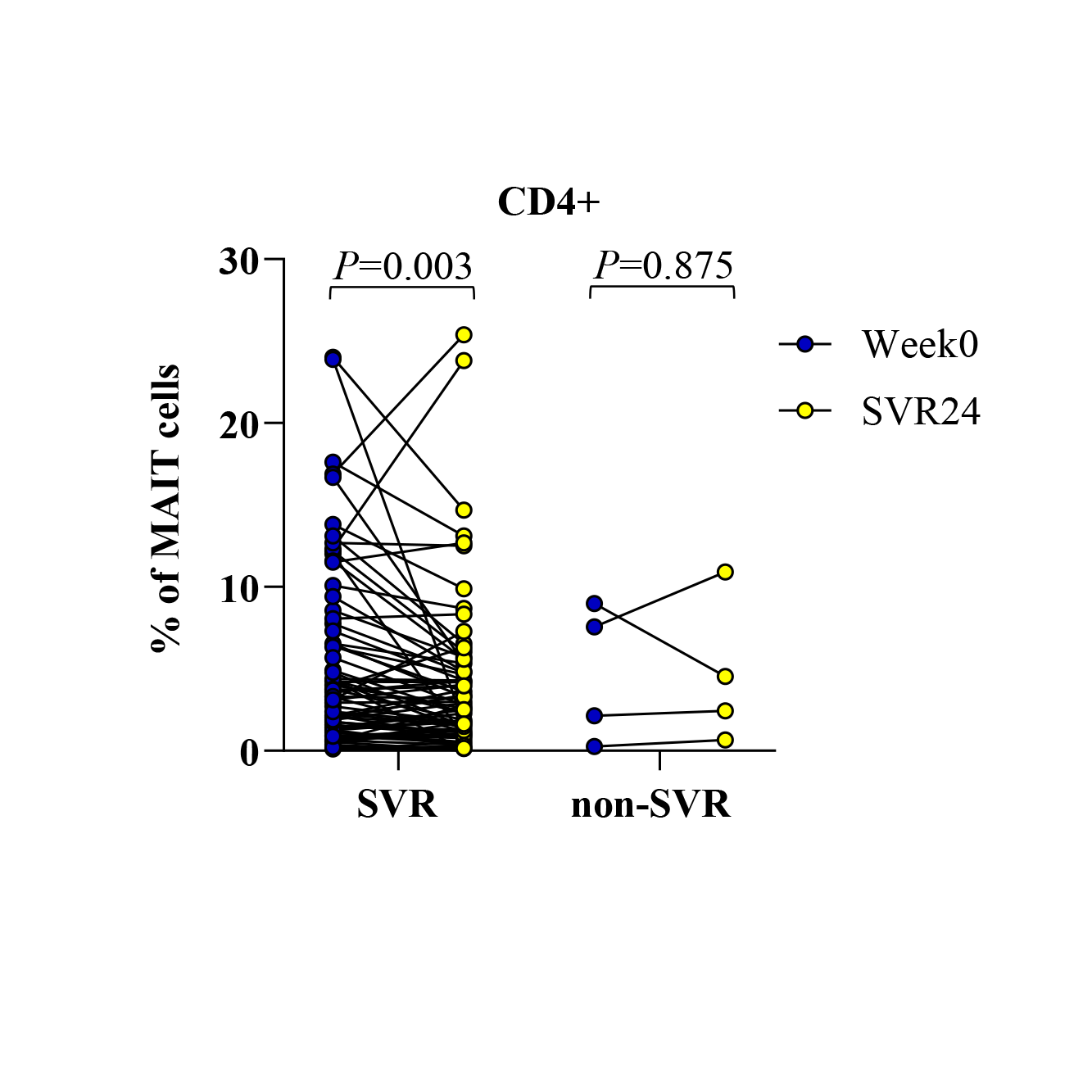

Supplement: S3 Fig — (TIF) [file pone.0244112.s003.tif]

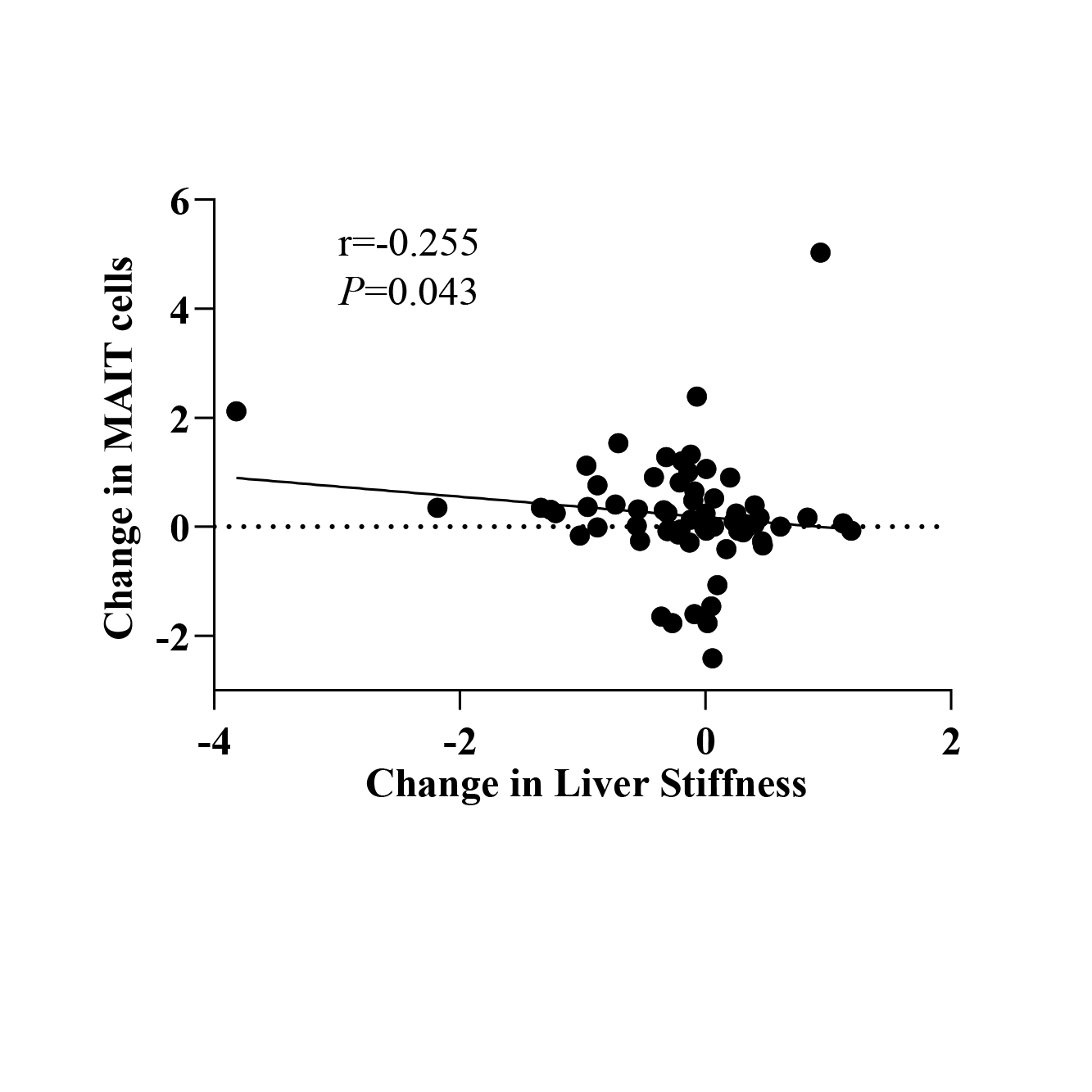

Supplement: S4 Fig — (TIF) [file pone.0244112.s004.tif]
